# Face Transplantation: On the Verge of Becoming Clinical Routine?

**DOI:** 10.1155/2014/907272

**Published:** 2014-06-09

**Authors:** Ralf Smeets, Carsten Rendenbach, Moritz Birkelbach, Ahmed Al-Dam, Alexander Gröbe, Henning Hanken, Max Heiland

**Affiliations:** Department of Oral and Maxillofacial Surgery, University Medical Center Hamburg-Eppendorf, Martinistraße 52, 20246 Hamburg, Germany

## Abstract

*Introduction*. Face transplantation (FT) is an innovative achievement of modern reconstructive surgery and is on the verge of becoming a common surgical opportunity. This review article was compiled to provide an update on this surgical field, especially regarding clinical outcomes, benefits, and complications implied.* Methods*. We performed an extensive research on all English-language Medline articles, case reports, and reviews published online until September 15, 2013. Used search terms were “face transplantation,” “face transplant,” “facial transplantation,” “facial transplant,” “face allograft,” and “facial allograft.”* Results*. To date 27 FTs have been performed worldwide. 19 of these cases have been published in the Medline database. Long-term follow-up reports of FT cases are rare. Three deaths associated with the procedure have occurred to date. The clinical outcomes of FT are satisfying. Reinnervation of sensation has been faster than motor recovery. Extensive functional improvements have been observed. Due to strict immunosuppression protocols, no case of hyperacute or chronic rejection and no graft-versus-host disease have occurred to date.* Conclusions*. As studies on long-term outcomes are missing, particularly regarding immunosuppression-related complications, FT will stay experimental for the next years. Nevertheless, for a small group of patients, FT already is a feasible reconstructive option.

## 1. Introduction

The reconstruction of acquired facial defects and deformity resulting from trauma, burns, and tumor resection has always been a great challenge for surgeons as the head and neck region provides complex anatomy and essential functions decisively contributing to each patient's quality of life. Surgical intervention should ideally result in both functionally and aesthetically satisfying results not last because the face contributes to each patient's identity formation and as this part of the body is always exposed, thus providing the basis for social interaction.

As conventional surgical procedures such as local or free flap transfers do not comply with these requirements in cases of extensive damage or loss of greater parts of the face involving multiple tissues, innovative therapeutic strategies had to be developed. Despite the fact that intensive discussions on dreaded and substantiated advantages and disadvantages had been held since 2002 [1–6], face transplantation (FT) has become this viable alternative.

FT is part of the vascularized composite tissue allotransplantation (VCA) concept referring to all nonorgan transplants and combining bone, tendons, muscles, nerves, vessels, and skin. It couples the principles of microsurgical reconstruction with those of human organ transplantation [7] and has thereby opened new possibilities in the reconstruction of severely disfigured patients [8]. Since the first FT was successfully performed in 2005 [9] at least 26 procedures followed in 7 countries and this new surgical field seems to mark transition from an experimental status to a common standard procedure. However, as severe complications including the decease of three patients have been observed and as there are only few data regarding long-term follow-up, a new debate has been raised [10].

This review article was compiled to provide an extensive overview and update on the world's experience with facial transplantation. The main outcome of the study was to identify the peculiarities and commonalities of all reported FT cases until 2013, with special regard to the clinical outcome and complications implied. Specific aims of this paper were to analyze preoperative, surgical, and logistic aspects as well as the clinical and psychological outcome of all FT cases in dependence of these surgical considerations. In particular we addressed the question of whether the kind of performed nerve repair had an influence on the time span until sensor recovery reoccurred. Further the study aimed to analyze the immunological protocols that were used.

## 2. Material and Methods

**Figure 1:**
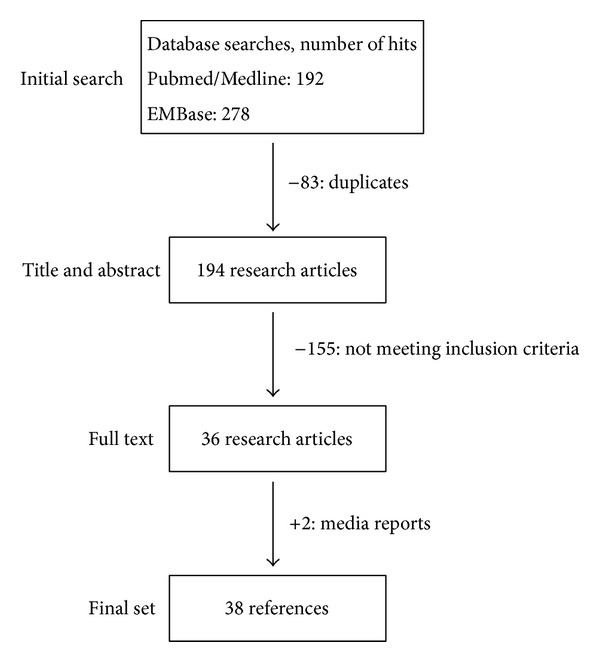
Flow diagram of the inclusion process.

In order to generate this retrospective review article on the current status and clinical outcomes of facial transplantation, we first performed an extensive literature research on Medline (http://ncbi.nih.gov/Pubmed) and on EMBase (http://elsevier.com/online-tools/embase). Search terms used included* face transplantation*,* face transplant*,* facial transplantation, facial transplant, face allograft,* and* facial allograft* published online until September 15, 2013. Only English-language articles on humans with an abstract provided were listed. This initial research generated 192 articles on Medline and 278 articles on EMBase. First of all, duplicates were removed. All entries were then screened by two authors for relevance according to our predefined inclusion and exclusion criteria (Table 1). Only articles containing information of at least one FT recipient or FT procedure regarding indications, harvesting procedure, microsurgical aspects, clinical and psychological outcomes, and therapeutic or immunosuppressive strategies were included in the compilation of our review. Only case reports, original articles, and review articles were taken into consideration. According to our inclusion and exclusion criteria, finally 36 articles were included in the compilation of our review [8, 9, 11–44]. Additionally information was gathered from scientific meeting presentations and from media reports, especially those concerning the latest cases with a follow-up period of less than one year. The concept of our literature search is provided in Figure 1.

In order to create an overview on the differences and peculiarities of the single FT cases, all articles were systematically screened for the outcome of interests listed in Tables 2, 3, 4(a, b), and 5. Data extraction of each article was performed by two of the primary authors. Each article was analyzed twice and uncertainties and the risk of bias of individual articles were addressed by discussion with the senior authors. Relevant data was finally listed in an Excel table for comparison and measurements. Principal summary measures were donor and recipient age difference in means, surgical duration on average, follow-up period on average, and sensory and motor recovery in months in dependence on the kind of performed nerve repair. No additional analyses were performed.

## 3. Results and Discussion

### 3.1. Main Characteristics

As far as reported 27 face transplantations have been performed to date in France (*n* = 9), the United States of America (*n* = 7), Turkey (*n* = 5), Spain (*n* = 3), China (*n* = 1), Belgium (*n* = 1), and Poland (*n* = 1). 19 of these cases have been published on Medline. Based on the number of cases, most experience exists at the University Hospital Henri Mondor, Créteil, France (Lantieri et al.), and at Brigham and Women's Hospital, Boston, USA (Pomahac et al.). Tables 2, 3, 4 and 5 provide an overview on the main characteristics of all procedures. The average age of the recipients was 34.6 ± 10.0 years, ranging from 19 to 59 years. The average age of the donors was 39.7 ± 13.0 years (19 to 65 years). Age disparity between donor and recipient varied from 1 year to 36 years. Eight recipients were more than 10 years younger than their corresponding donor. The recipient male-to-female ratio was 21 : 5. To date there are 3 deaths associated with the procedure, representing 11.1% of all recipients. One patient died 27 months after surgery because of a lack of compliance considering the immunosuppressive therapy [10]. The second case of death has occurred in the third French recipient due to prolonged anoxic cardiac arrest following multidrug-resistant infections and consequent graft necrosis [11]. The third patient deceased in July 2013 after suffering from recurrent cancer [45].

To date, the 14 different surgical teams harvested 16 partial and 10 full facial allografts. As far as published there were at least 10 myocutaneous transplantations, whereas at least 15 explants included bone. Other tissues like septal cartilage and the parotid or lacrimal glands were transplanted in as many as 11 cases. There were at least 2 allografts that included the tongue (Table 3). So far, no case of graft loss, hyperacute or chronic rejection, or graft-versus-host disease has been reported. With exception of one case, all patients who underwent facial transplantation surgery returned to normal life [8, 11].

### 3.2. Indications and Recipient and Donor Selection

The inclusion criteria for FT programs vary from center to center. To date only patients with extensive tissue damage resistant to conventional reconstruction procedures have been included. Lantieri and his team considered only defects including full destruction of orbicularis oris and/or orbicularis oculi muscles to be without a prospect of a successful reconstruction by conventional means [12]. The team in Boston only included patients with a defect comprising >25% of the facial area and/or loss of one of the central facial parts such as eyelids, nose, or lips [13]. In order to find the optimal candidates for FT, Siemionow et al. developed a preliminary assessment tool called the FACES score [14]. Patients were excluded in case of significant medical comorbidity, missing guarantee for posttransplant follow-up, high risk of recurrent cancer under immunosuppression, and pregnancy [13, 15]. Protocols for FT provided only psychologically and immunologically stable patients as potential recipients [15].

Since the first full facial allograft was successfully transplanted in 2010 [16], the group of potential candidates has expanded. To date, face transplantation has been performed on patients with severe burns (*n* = 10, including chemical and electrical burns), gunshot injuries (*n* = 8), animal attacks (*m* = 3), neurofibromatosis type I (*n* = 3), severe radiotherapy side effects, and crush trauma (*n* = 1) (Table 2). Before going into the relevant transplant programs, all recipients had undergone various surgeries for reconstruction, each of them with poor functional and aesthetic outcome [9, 12, 16–22, 44]. Donors were selected based on race, skin color, sex, blood type, age, HLA matching, and immunological status [12, 16–19, 22].

### 3.3. Harvesting Procedure

Cold ischemia time is one of the most important aspects affecting the success rate in solid organ transplantation. Following cardiac arrest caused by infusion of specified organ preservation solutions, fast harvesting, transport, and implantation are required. As far as reported, most facial allografts were harvested from brain-dead heart beating donors. In order to reduce cold ischemia time the surgical teams prepared their corresponding harvesting procedure in case of multiorgan procurements by dissection of greater parts of the face under maintenance of circulation before cross clamp time, so the final surgical steps hereafter could be managed within a short period of time [15].

In order to reduce tissue damage and to avoid blood congestion during cold ischemia time, some surgical teams opted for allograft perfusion and storage in different organ preservation solutions at 4°C as known from solid organ transplantation. As far as reported only ILG-1 (Institut Georges Lopez Organ Preservation Solution) and UWC (University of Wisconsin Solution) were used in this context [9, 16, 19].

Restoration of the donor's facial appearance following the removal of the allograft is one important issue, especially regarding ethical concerns and psychological burden of the donors' families. As far as reported restoration was mostly performed via construction of painted resin masks following alginate molding of the donors' faces preoperatively [17, 21–23].

### 3.4. Surgical Considerations

Facial allografts have varied in the composition of the involved tissues, conditioning the extent of the respective surgical procedure. As far as reported transplantation of bone, requiring open fixation, has been performed in at least 12 cases, most of which contained the mandible and/or maxilla including teeth. Most of the transplants included cheeks, nose, eyelids, and lips, whereas the parotid gland and septal cartilage have been transferred in 10 cases (Table 3).

Despite the complexity of the procedure, no surgical failure has been reported to date, which might be explained by good circulation in the head and neck region and the highly skilled surgeons in the respective transplant centers. Early cases and cadaver studies have demonstrated that transplant perfusion may generally be achieved with few vascular anastomoses [9, 19, 20, 24]. Moreover it has been shown that revascularization of the whole face and maxilla can be achieved by connection of one single facial artery [16, 25]. In order to reduce the risk of postoperative thrombosis and potential transplant loss, most anastomoses were performed in large diameter vessels. Most surgeons opted for a bilateral connection of the facial or external carotid artery (Table 4(a)). Venous drainage was mostly ensured via end-to-end connection of the external jugular vein (*n* = 6), facial vein (*n* = 4), or junction to the thyrolinguofacial trunks (*n* = 4). With exception of two reported cases all anastomoses were performed using conventional end-to-end microsurgical techniques.

Besides creating vascular anastomoses and in order to generate an optimal functional outcome, microsurgical skills are also needed for motor and sensory neurorrhaphies. Concerning the facial nerve, different approaches have been used. Teams dissected the facial nerve directly behind the stylomastoid foramen, performing neurorrhaphy following superficial parotidectomy [26]. Alternatively only single branches of the facial nerve distal to the parotid gland were connected [9, 42]. The Spanish team led by Cavadas even performed connection of the hypoglossal nerve [20].

Finally, with the objective of full sensory nerve recovery, most transplant teams connected the infraorbital and mental nerve, whereas neurorrhaphy of the supraorbital nerve has been performed in 5 cases (Table 4(a)). In one case each, connection of the buccal sensory nerve [23] and of the lingual and infra-alveolar nerve [20] has been performed. In 4 cases sensory nerves were only placed near the corresponding nerve exit point (Table 4(a)).

As far as reported, transplantation of the human face, including anatomical structures like cartilage and bone and requiring microsurgical anastomoses of nerves and vessels as well as multiple osteosynthesis, took an average of 17.6 hours (Table 2). To complete the procedure extensive planning and coordination of different surgical teams were necessary. As face transplantation has not been included in any national standard transplantation organization like Eurotransplant, donor selection had to be performed individually and donor family consent had to be obtained.

### 3.5. Clinical Outcome

The establishment of face transplantation as a surgical procedure in 2005 marked a turning point in the history of reconstructive surgery and gave new hope to severely disfigured patients possibly meeting both requirements. Eight years after Devauchelle and Dubernard successfully transplanted the first human face, clinical results are satisfying. Sensory recovery has been observed as early as 12 weeks postoperatively, with regaining temperature perception later than tactility [12, 19, 26]. The time of recurrence of sensation was thereby dependent on the fact whether sensory nerve coadaptation had been performed or not (Table 4(b)). As far as reported at least 12 patients regained aesthesia within the first year following transplantation [27] including cases without repairing branches of the trigeminal nerve due to the extent of damage [12, 18, 19].

As expected and after 7.8 months on average, motor recovery has occurred later than sensory recovery (Table 4(b)). As far as reported 12 patients regained their motor functions with varying success rates regarding early results. First contractions of single muscles have been observed as early as 2-3 months after surgery [12] while complex movements appeared within the first year [12, 16, 17, 19, 23, 26, 27]. The recipient of the first near total FT was given back the ability to smell, drink from a cup, and eat solid food within a very short period of time following surgery. After 5 years the clinical results of the first FT patient exceeded expectations with the recipient being able to fully open her mouth, smile, speak, chew, and swallow again [28]. As long-term results are still pending, a final assessment regarding sensomotorical outcomes cannot be made presently.

### 3.6. Psychological Outcome

Face transplantation is a challenge for the recipients and there has always been an intense debate on potential psychological distress, comorbidity, and the correct patient oriented management around this procedure [6, 29, 30]. Contrary to the fears expressed, up to now no evidence for problems regarding identity crises and body image changes was described. In fact, there are several reports on good psychological outcomes with recipients accepting their new facial appearances accompanied by fast social reintegration [9, 16, 19, 31]. To date three published reports on quantitative psychological testing have been published. Regarding three recipients, Chang and Pomahac found significant improvement on quality of life measures of physical and mental health based on the MOS-SF 12 [32]. Using different quantitative and nonquantitative, subjective quality of life assessments like the Short Form questionnaire 36 (SF-36) and the Derriford Appearance Scale 59 (DAS-59), Lantieri and his team showed quantitative improvements in the quality of life of his first patients, respectively. Three of his patients even returned to work, a fact facilitating their social reintegration [13]. Finally Coffman and Siemionow suggested the introduction of a standard psychological follow-up protocol for further examination after transplant surgery, raising the question of whether a significant improvement of quality of life may offset the risks and side effects of immunosuppression [33].

### 3.7. Immunological Aspects

Allograft rejection, which is due to genetic incompatibility between the components of the donor and recipient tissue, has always been a serious issue in organ transplantation [34]. Since rejection may occur at different times after transplantation, it has been classified as hyperacute (within the first 48 hours), acute (within days and months after transplantation), or chronic [35]. Because skin and oral mucosa are well known to have high immunogenic property and often comprise the largest part of a composite facial allograft, rejection is expected to be particularly problematic. Although no hyperacute graft rejection has been reported so far, acute rejections seem to be inevitable: each of the FT recipients who had a follow-up period of more than one year had at least one episode of acute rejection [8], manifesting itself in reddening of the skin, swelling, and the presence of nodules and papules. However, the estimated incidence of 30–50% of chronic rejection, which had been expected to occur within 5 years after face transplantation, has not been proven true so far [6]. The first face transplant patient is now 8 years after surgery and fortunately to date there has been no reported case of chronic rejection and graft-versus-host disease [28].

Acute rejections could successfully be treated with increased immunosuppressive therapy [21]. In order to avoid allograft rejection, all teams performed blood type screening and HLA matching preoperatively. In various cases, a sentinel free flap from the donor was transplanted for surveillance biopsies and monitoring the clinical and pathologic signs of graft rejection [23, 36].

Similar to regimen used in solid organ transplantation, immunosuppressive therapy in patients with a facial allograft consists of an “induction,” started in an early stage during or even before surgery, and “maintenance” for as long as the transplant remains in the recipient. Although strategies of immunosuppression diversify slightly from center to center, most of the face transplantation groups employed an induction therapy with polyclonal antithymocyte globulins (ATG), anti-interleukin-2 (IL-2) receptor monoclonal antibodies such as daclizumab and basiliximab, anti-CD3 monoclonal antibodies, mycophenolate mofetil, methylprednisolone, and the calcineurin-inhibitor tacrolimus. Maintenance was typically a triple therapy comprising tacrolimus, mycophenolate mofetil, and prednisolone [37–41].

Lately, there has been increasing interest in finding alternative strategies to standard immunosuppressive therapy in order to avoid the problems associated with its serious side effects. The ultimate aim is an ideal status of immune tolerance, which is when the recipient of a transplant tolerates the allograft in the absence of immunosuppression. The most promising current approaches in research focus on the development of anti-T-cell antibodies, extracorporeal photopheresis, and stem cell therapy [41].

### 3.8. Complications and Future Directions

Despite the complexity of the procedure there are no reports on transplant loss due to surgical failure such as arterial or venous thrombosis or tissue damage due to prolonged cold ischemia time. However the first recipient of a full face allograft in Spain developed a thrombosis on postoperative day 3 that could be managed successfully by surgical reintervention [16]. Further common surgical complications, wound healing disorders, stenosis of Stensen duct, postoperative ptosis, ectropion of eyelids, bleeding, and pain occurring in various cases could all be managed by conservative or surgical intervention [8].  Table 5 provides an overview on all reported complications due to FT procedures to date.

The most important complications due to face transplantation programs are those arising from immunosuppressive therapy including drug toxicity leading to metabolic disorders, opportunistic infections, and increased incidence of malignancy. Tacrolimus especially, a potent calcineurin inhibitor, is known for its serious nephrotoxicity [12]. In spite of rigorous antimicrobial prophylactic medication protocol, the majority of patients with facial allografts have suffered from opportunistic infections, including cytomegalovirus, herpes simplex, herpes zoster,* Candida albicans*, and bacterial infections, most of which could be treated successfully [36]. Due to an increased risk of carcinogenesis in the context of a suppressed immune system, it is most likely that a correlation exists between the immunosuppressive medication and occurrence of malignomas in FT patients. In this context the first FT recipient has been diagnosed with cervical dysplasia, which had to be removed surgically [42]. An Epstein-Barr virus infection may have caused a B-cell lymphoma in the second patient of the Dubernard/Devauchelle group but could be successfully treated with rituximab [46]. Finally patient 9 was diagnosed with recurrent squamous cell carcinoma under immunosuppression. In this respect neurofibromatosis type 1 as an indication for facial transplantation should be critically questioned, as the development of malignant peripheral nerve sheath tumors may occur under immunosuppression, as already observed following solid organ transplantations [43].

Finally, three cases of death have been reported to date. The first patient not to survive the procedure received concomitant face and bilateral hand transplantations. He developed a multiresistant* Pseudomonas aeruginosa* infection of the allograft which led to a prolonged intensive care therapy with septic shock and pneumonia which consecutively led to death [45]. In this regard the combined extremity and face transplantation has to be critically discussed, as there have been severe complications leading to extremity transplant loss in the second combined allograft patient [44]. The second death in the FT history occurred in the Chinese recipient who died 27 months after the operation. The exact cause of his death has not been fully elucidated, but it is believed that this was because of incompliance with the immunosuppressive therapy, causing multiorgan failure [42]. The third patient deceased due to an aggressive recurrent squamous cell carcinoma of the tongue under immunosuppression and a predominant HIV infection. However medical details on the circumstances have not yet been reported [47].

With the third casualty due to face transplantation the importance of candidate selection as one key determinant of success again has been approved. The indication to include patients with predominant cancer or facial defects due to cancer or oncologic therapies in the patients' medical history should be reconsidered carefully. With FT presenting a rather life-enhancing than life-saving procedure and the potential risks due to immunosuppression, patients should only be considered as candidates in case of an intact immune system.

After a decade of experience the surgical components of facial transplantation are mostly clarified. Clinical and psychological results are satisfying and to date there has been no case of transplant loss. However the risk of a potential transplant loss due to chronic rejection stays remarkable and to date it is still unclear which treatment options would be applicable in this particular case. As long-term results showing clinical, psychological, and immunological outcomes in dependence of different surgical considerations and immunological protocols after 5, 10, or 20 years are mostly missing, a final evaluation of FT may not be done. Face transplantation will not be considered as a routine practice, until the rate of complications due to this procedure is remarkably lowered and until long-term reports approve long-term survival of allografts and the patients. Thus future research directions should and will focus on induction of transplant tolerance, where recipients do not require long-term immunosuppression. Although VCAs including skin are considered the most antigenic of all tissues, there are already promising studies in this field [48]. Furthermore alternative strategies for the reconstruction of severely disfigured patients should be fathomed.

## 4. Conclusion

With the experience of 27 cases to date, demonstrating the feasibility of the procedure, face transplantation seems to have become a viable reconstructive option for severely disfigured patients. However, with exception of the first case, there is a lack of mid- and long-term reports analyzing functional, psychological, and aesthetic outcomes after a longer period of time, especially regarding complications. Although early results are encouraging, there is still a lot of work to do until completion of this procedure could generally be recommended. The pioneers and newcomers in this surgical field should pool together their results and try to develop guidelines and standard protocols for the implementation of the procedure. Evaluating the chances and optimizing the process of FT might be easier, if all recipients were included in standardized follow-up protocols, making it possible to compare the results and to work out unsolved problems with the objective of improving knowledge. Regarding the immunosuppressive therapy, research has to be done and alternative protocols should be tested. As long as the occurrence of side effects and complications due to the immunological therapy remains high and as long as the risk of transplant loss after many years is not lowered or certainly ruled out, facial transplantation will stay experimental, leaving surgeons and patients in uncertainty on the final outcome.

Despite eight years of experience facial allografts are still an exceptional reconstructive option that should only be considered for certain cases. In the long term, face transplantation may be replaced by more compatible reconstructive options reducing the risks and improving the results, possibly based on tissue engineering principles.

## Figures and Tables

**Table 1:** Inclusion and exclusion criteria of the literature research.

Inclusion criteria	Exclusion criteria
*✓* English-language papers	*✓* Cadaver study
*✓* Human study	*✓* Anatomical study
*✓* Case reports	*✓* Experimental study
*✓* Original articles	*✓* Brief communications
*✓* Review articles	*✓* Letters and comments

**Table 2:** Main characteristics of the first 27 face transplantations.

Number	Year	City & team leader	R sex, age	D age	Indication	Type	SD (h)	FUP (y)
1	11/2005	Devauchelle Amiens, France	F, 38	46	Animal attack	Partial myocutaneous	15	7.8
2	04/2006	Guo Xian, China	*M, 30	25	Animal attack	Partial osteomyocutaneous	13	7.4
3	01/2007	Lantieri Paris, France	M, 29	65	NF 1	Partial myocutaneous	11	6.8
4	12/2008	Siemionow Cleveland, USA	F, 45	44	Gunshot injury	Partial osteomyocutaneous	22	4.8
5	03/2009	Lantieri Paris, France	M, 27	43	Gunshot injury	Partial osteomyocutaneous	19	4.6
6	04/2009	Lantieri Paris, France	*M, 37	59	Burns	Partial myocutaneous	13	4.5
7	04/2009	Pomahac Boston, USA	M, 59	60	Burns	Partial osteomyocutaneous	17	4.5
8	08/2009	Lantieri Paris, France	M, 33	55	Gunshot injury	Partial osteomyocutaneous	16	4.2
9	08/2009	Cavadas Valencia, Spain	M, 42	35	Radiotherapy	Partial osteomyocutaneous	15	4.2
10	11/2009	Devauchelle Amiens, France	M, 27	—	Burns	Partial osteomyocutaneous	19	3.9
11	01/2010	Gomez-Cia Seville, Spain	M, 35	30	NF 1	Partial myocutaneous	22	3.8
12	04/2010	Barret Barcelona, Spain	M, 31	41	Gunshot injury	Full osteomyocutaneous	—	3.5
13	07/2010	Lantieri Paris, France	M, 37	—	NF 1	Full myocutaneous	14	3.3
14	03/2011	Pomahac Boston, USA	M, 25	48	Burns	Full myocutaneous	17	2.5
15	04/2011	Lantieri Paris, France	M, 45	—	Gunshot injury	Partial osteomyocutaneous	—	2.4
16	04/2011	Lantieri Paris, France	M, 41	—	Gunshot injury	Partial osteomyocutaneous	—	2.4
17	04/2011	Pomahac Boston, USA	M, 30	31	Burns	Full myocutaneous	14	2.4
18	05/2011	Pomahac Boston, USA	F, 57	42	Animal attack	Full osteomyocutaneous	19	2.3
19	01/2012	Özkan Antalya, Turkey	M, 19	39	Burns	Full osteomyocutaneous	9	1.7
20	01/2012	Blondeel, Gent, Belgium	N/A	N/A	N/A	Partial osteomyocutaneous	20	1.7
21	02/2012	Nazir Ankara, Turkey	M, 25	40	Burns	Full face transplant	—	1.6
22	03/2012	Özmen Ankara, Turkey	F, 20	28	Burns	Partial face transplant	—	1.5
23	03/2012	Rodriguez Baltimore, USA	M, 37	21	Gunshot injury	Full osteomyocutaneous	36	1.5
24	05/2012	Özkan Antalya, Turkey	M, 27	19	Burns	Full face transplant	—	1.3
25	01/2013	Pomahac Boston, USA	F, 44	—	Burns	Full myocutaneous	15	0.6
26	05/2013	Özkan Antalya, Turkey	M 27	19	Gunshot injury	Partial osteomyocutaneous	—	0.5
27	07/2013	Maciejewski Warsaw, Poland	M, 33	42	Crush trauma	Partial osteomyocutaneous	27	0.3

R = recipient, D = donor, D = difference, SD = surgery duration, FUP = follow-up period, and ∗ = patient died.

**Table 3:** Transplanted tissues (25 reports to date).

Myocutaneous		Osseous		Other	
Mucosa	25	Maxilla	8	Parotid gland	10
Nose	24	Maxilla	2	Septal cartilage	9
Cheeks	23	Nasal bone	3	Tongue	2
Upper lip	23	Zygomatic bone	2		
Lower lip	19	Orbital floor	2		
Chin	16				
Lower eyelid	11				
Upper eyelid	10				
Forehead	10				

**Table:** (a)

Arterial anastomosis		Venous anastomosis		Sensory nerve repair		Motor nerve repair	
External carotid artery	12	External jugular vein	6	Infraorbital nerve	10	Facial nerve	15
Facial artery	6	Facial vein	4	Mental nerve	8	Hypoglossal nerve	1
External maxillary artery	1	Internal jugular vein	5	Supraorbital nerve	5	Mandibular branch	1
CCA to SCA	1	Thyrolingual trunks	4	None	5		
		Retromandibular vein	3				
		Anterior facial vein	2				

**Table:** (b)

Sensory recovery (months after transplantation)			Motor recovery (months after transplantation)	
Nerve repair	Touch	Temperature	Nerve repair	Motor recovery

Yes	4.1	4.5	YES	7.8
No	7.3	12.5	NO	—

**Table 5:** Complications reported in face transplant recipients.

Immunotherapy complications	*n*
Infections	
Viral (CMV, HSV, EBV, and Poxyvirus)	8
Bacterial (*Pseudomonas*, staphylococcal, and other)*	7
Fungal (*Candida*)	3
Rosacea	1
Metabolic	
Acute rejections	14
Renal failure	2
Glucose intolerance/diabetes mellitus	2
Transient leukopenia	1
Severe rhabdomyolysis	1
Neoplasia	
Cervical dysplasia	1
Monoclonal B-cell lymphoma	1
Secondary squamous cell carcinoma*	1

Other complications	*n*

Acute respiratory distress syndrome	1
Right diaphragmatic paralysis	1
Transient thrombocytosis	1
Thrombotic microangiopathy	1

*Complication leading to death.

*n* = number.
